# Supported Ionic Liquid Membranes and Ion-Jelly^®^ Membranes with [BMIM][DCA]: Comparison of Its Performance for CO_2_ Separation

**DOI:** 10.3390/membranes5010013

**Published:** 2015-01-14

**Authors:** Ricardo Couto, Luísa Neves, Pedro Simões, Isabel Coelhoso

**Affiliations:** REQUIMTE, Departamento de Química, Faculdade de Ciências e Tecnologia, Universidade Nova de Lisboa, 2829-516 Caparica, Portugal; E-Mails: rmc09551@campus.fct.unl.pt (R.C.); lan11892@fct.unl.pt (L.N.); pcs@fct.unl.pt (P.S.)

**Keywords:** supported ionic liquid membranes, Ion-Jelly^®^ membranes, CO_2_ separation, ionic liquids

## Abstract

In this work, a supported ionic liquid membrane (SILM) was prepared by impregnating a PVDF membrane with 1-butyl-3-methylimidazolium dicyanamide ([BMIM][DCA]) ionic liquid. This membrane was tested for its permeability to pure gases (CO_2_, N_2_ and O_2_) and ideal selectivities were calculated. The SILM performance was also compared to that of Ion-Jelly^®^ membranes, a new type of gelled membranes developed recently. It was found that the PVDF membrane presents permeabilities for pure gases similar or lower to those presented by the Ion-Jelly^®^ membranes, but with increased ideal selectivities. This membrane presents also the highest ideal selectivity (73) for the separation of CO_2_ from N_2_ when compared with SILMs using the same PVDF support but with different ionic liquids.

## 1. Introduction

Supported Ionic Liquid Membranes (SILMs) have attracted great attention in the past years, due to their ease of preparation and versatility. SILMs can be prepared by impregnating a porous membrane with an ionic liquid. Ionic Liquids (ILs) are regarded nowadays as valuable solvents that may in the short to mid-term replace conventional solvents in various applications. Ionic Liquids are compounds consisting entirely of ionic species, with an organic cation and an inorganic or organic anion. The physical and chemical properties of ILs may be tuned according to the cation and anion present in their structure, and one of their most important properties is that they present a negligible vapor pressure. In the case of membrane processes, ILs are being used in the design and modification of advanced materials that enable performance levels not typical of conventional materials [1]. In a SILM, the ionic liquid will improve the selectivity towards a specific compound, while the porous support provides an adequate membrane mechanical stability. Due to the large amount of flat sheet membranes and ionic liquids available, the possible combinations are countless.

Traditional supported liquid membranes based on organic solvents, present some limitations in stability due to the ease of displacement of the liquid phase from the membrane support, either due to dissolution or dispersion into the surrounding medium or through evaporation at high temperatures. Since ILs have negligible vapor pressure, an important advantage of using SILMs is that minimal membrane liquid loss through solvent evaporation is guaranteed, which allows to obtain more stable membranes due to the higher viscosity of ILs and greater capillary forces between the desired ionic liquid and the polymer membrane support [2].

SILMs have been used for a wide range of applications, including separation of organic compounds, pervaporation and vapor permeation, separation of ions, analytical applications, electrochemical applications and gases separations [3]. Specifically, SILMs may be appropriate for industrial applications in the separation of gases, such as the treatment of bio-methane from anaerobic digesters and CO_2_ capture from flue gases [4].

A broad diversity of ILs has already been tested for developing SILMs. Taking into account the solubility and selectivity of CO_2_ in ILs as well as their highly tunable nature, several studies on the permeability and selectivity properties of gases through SILMs have explored the effect of the IL structure [5]. Concerning the influence of the IL cation, it has been investigated the gas permeation properties of different families of ILs such as imidazolium [4,6,7,8,9,10,11], pyrrolidinium [12,13,14], pyridinium [15], ammonium [16], phosphonium [17,18], or cholinium [19] and improved results were obtained for imidazolium-based SILMs in terms of permeability and selectivity. Regarding the IL anion, the performance of imidazolium-based ILs containing several different anions such as bis(trifluoromethylsulfonyl)imide ([NTf_2_]^−^) [4,6], hexafluorophosphate ([PF_6_]^−^) [4,17], dicyanamide ([DCA]^−^) [6], among others, has been evaluated, and the results indicate that nitrile-containing anions promote an increase in both CO_2_ permeability and CO_2_/N_2_ selectivity.

One of the drawbacks of SILMs is the fact that ionic liquids can be displaced from the support matrix by pressure differences across the membrane as low as 0.2 MPa [4]. To avoid this problem, several authors have proposed to blend the IL with a polymer, creating a gel [20,21,22]. Membranes prepared in this way retain solubility characteristics identical to the liquid, which confer them identical permeabilities, but with improved mechanical properties. These membranes were effectively employed in the separation of gases such as carbon dioxide from nitrogen [20,22], methane [21,22], and hydrogen [22], or hydrogen from nitrogen [22]. In a previous work, gelatin was used as gelling material [23], originating a material known as Ion-Jelly^®^ [24,25,26,27,28], which was spread over a cellulose sheet to confer support for the membrane. It was shown that these membranes can sustain up to 10 MPa of pressure difference across the membrane without significant loss of ionic liquid, while presenting permeabilities to pure gases (H_2_, N_2_, O_2_, CO_2_ and CH_4_) similar to those obtained with SILMs using the corresponding IL, although with lower ideal selectivities.

In this work we have prepared a SILM based on a PVDF flat sheet membrane and 1-butyl-3-methylimidazoilum dicyanamide ([BMIM][DCA]) ionic liquid and studied its application as a membrane for the separation of gases (CO_2_, N_2_ and O_2_). Furthermore, we compare its performance, in terms of permeability and selectivity, with those obtained with Ion-Jelly^®^ membranes [23].

## 2. Results and Discussion

The permeabilities of CO_2_, N_2_ and O_2_ through the PVDF [BMIM][DCA] membrane as well as the calculated ideal selectivities for these gases are presented in Table 1. In Table 2 are presented some properties of the IL [BMIM][DCA] to help in the following discussion. The SILM presents a CO_2_ permeability two orders of magnitude higher than to the other gases, which may be due to its high solubility in [BMIM][DCA] when compared with the other gases [29]. Consequently, the calculated ideal selectivities are higher for separating CO_2_ from N_2_ or O_2_ than for separating O_2_ from N_2_, with the highest ideal selectivity being that between CO_2_ and N_2_.

**Table 1:** Permeabilities of pure gases and calculated ideal selectivities, including results for an Ion-Jelly^®^ membrane from a previous work [23].

Membrane	Permeability (barrer)		Ideal selectivity	
PVDF [BMIM][DCA]	CO_2_	145	CO_2_/N_2_	73
	N_2_	2	CO_2_/O_2_	21
	O_2_	7	O_2_/N_2_	4
IJ [BMIM][DCA] [23]	CO_2_	120	CO_2_/N_2_	10
	N_2_	12	CO_2_/O_2_	5
	O_2_	24	O_2_/N_2_	2

**Table 2:** Properties of [BMIM][DCA].

Physical property	Physical property value
Density	1.0555 g∙cm^−3^ (303.15 K) [32]
Viscosity	24.4 ± 0.2 mPa∙s (303.15 K) [32]
Surface Tension	48.6 ± 0.1 mN∙m^−1^ (303.15 K) [32]
CO_2_ solubility in IL	0.015 mole fraction (0.113 MPa; 323.15 K) 0.024 mole fraction (0.177 MPa; 323.15 K) [29]
Equilibrium solubility in water	12 g/L

Table 1 also presents the results obtained in a previous work [23] for an Ion-Jelly^®^ membrane prepared using the same ionic liquid ([BMIM][DCA]), for comparison. We can observe that the PVDF SILM presents lower N_2_ and O_2_ permeability values than the Ion-Jelly^®^ one. This difference may be due to the large amount of water in the Ion-Jelly membrane^®^ when compared with the SILM. Regarding the ideal selectivity results, the presence of water induces a less selective microenvironment for the transport of gases, and as a result a lower ideal selectivity for the gases in the Ion-Jelly^®^ membrane was observed.

**Figure 1:**
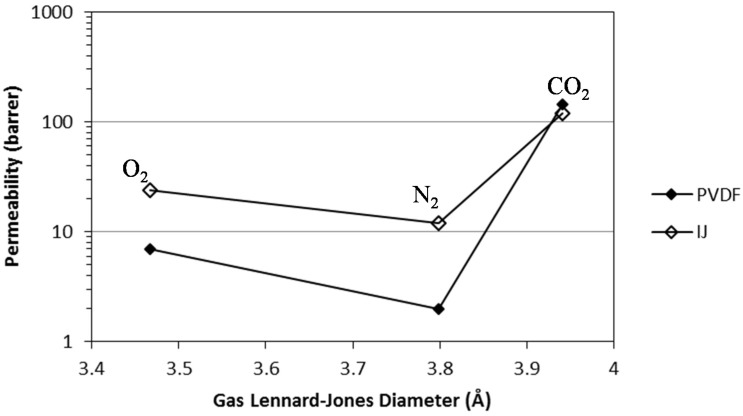
Comparison of the gas permeabilities of the PVDF and Ion-Jelly^®^ [23] membranes as a function of the gas Lennard-Jones diameter.

In Figure 1 are plotted the gas permeabilities against the Lennard-Jones diameter of the gas molecules. We can observe that the PVDF SILM presents the same trend in permeability as that previously observed for the Ion-Jelly^®^ membrane, with O_2_ presenting a higher permeability than N_2_, due to its smaller size, while CO_2_ presents the highest permeability value, despite its larger size, due to its high solubility in the ionic liquid used.

Furthermore, the results obtained in this work follow the same trend observed previously by Neves *et al.* for the PVDF SILMs made from 1-butyl-3-methylimidazolium hexafluorophosphate ([BMIM][PF_6_]), 1-octyl-3-methylimidazolium hexafluorophosphate ([OMIM][PF_6_]), 1-butyl-3-methylimidazolium tetrafluoroborate ([BMIM][BF_4_]), 1-butyl-3-methylimidazolium bis(trifluoromethanesulfonyl)imide ([BMIM][NTf_2_]) and 1-decyl-3-methylimidazolium tetrafluoroborate ([DMIM][BF_4_]) [4]. When comparing the ideal selectivities between CO_2_ and N_2_ obtained in both works, it is possible to observe that [BMIM][DCA] presents the highest ideal selectivity of all the ILs tested (Table 3). Even though a higher Henry’s constant for CO_2_ is obtained for this IL than in the other ones tested previously (4.87 MPa for [BMIM][NTf_2_] [30], 7.44 MPa for [BMIM][DCA] [29], at 323K), which corresponds to a lower solubility value, the CO_2_ diffusion coefficient is higher. Morgan *et al.* [9] developed a correlation for the diffusivity of gases in imidazolium ionic liquids at 303.15 K which is shown in Equation (1):

(1) $$D_{12}=2.66\times 10^{-3}\frac{1}{\mu_{2}^{0.66\pm 0.03}{\bar{V}}_{1}^{1.04\pm 0.08}}$$

where *D*_12_ is the diffusivity of gases in RTILs (cm^2^∙s^−1^); *µ*_2_ is the RTIL viscosity (mPa∙s) at 303.15 K; and *V*_1_ is the gas molar volume (cm^3^∙mol^−1^). Using this correlation, the diffusivity coefficients for CO_2_, O_2_ and N_2_ in [BMIM][DCA] and [BMIM][NTf_2_] were obtained and are presented in Table 4. It can be noticed that they are always higher for BMIMDCA, due to its lower viscosity.

**Table 3:** Ideal selectivities obtained in this and in a previous work [4].

RTIL	Ideal Selectivity CO_2_/N_2_
[BMIM][DCA]	73
[BMIM][PF_6_] [4]	23
[BMIM][BF_4_] [4]	35
[BMIM][NTf_2_] [4]	39
[OMIM][PF_6_] [4]	23
[DMIM][BF_4_] [4]	22

**Table 4:** Diffusivity coefficients for CO_2_, O_2_ and N_2_ in [BMIM][DCA] and [BMIM][NTf_2_], calculated using Equation (1).

Ionic Liquid	D_CO_2__ (cm^2^/s)	D_N_2__ (cm^2^/s)	D_O_2__ (cm^2^/s)
[BMIM][DCA]	6.97 × 10^−6^	8.00 × 10^−6^	1.05 × 10^−5^
[BMIM][NTf_2_]	3.94 × 10^−6^	4.52 × 10^−6^	5.70 × 10^−6^

Comparing the results available in the literature for the SILM based on the same IL ([BMIM][DCA]), Jindaratsamee *et al.* [31] obtained a permeability value of 200 barrer for CO_2_ and an ideal selectivity of 78.9 for the CO_2_/N_2_ separation, values which are similar to ours in the case of the ideal selectivity but higher in the case of the permeability. This difference in permeability could be a result of differences in the water content of the ionic liquids used to prepare the membranes or in the quantity of IL effectively impregnated in the membrane.

## 3. Experimental Section

**Figure 2:**
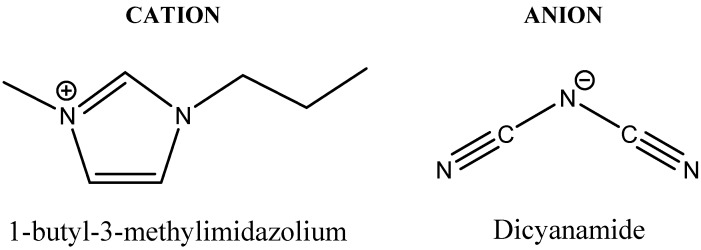
Structure of ionic liquid used in this work.

Ionic liquid 1-butyl-3-methylimidazolium dicyanamide ([BMIM][DCA]) was purchased from Iolitec (Heilbronn, Germany, 99% purity). The structure of this ionic liquid is presented in Figure 2.

The gases used in the experiments were oxygen (high-purity grade (99.999%), Praxair, Danbury, CT, USA), nitrogen (industrial grade (99.99%), Praxair, Danbury, CT, USA), and carbon dioxide (high-purity grade (99.998%), Praxair, Danbury, CT, USA). The PVDF flat sheet membrane used in this work was supplied by Millipore Corporation, USA, and was hydrophobic, with a porosity of 0.75, pore size of 0.22 µm and thickness of 125 μm.

### 3.1. Preparation of Membranes

A disk was cut from PVDF flat sheet membrane, and was placed in the bottom of a high pressure stainless steel vessel. 1 mL of [BMIM][DCA] ionic liquid was spread on top of the membrane, and the vessel was closed. CO_2_ was added to the vessel, at a pressure of 0.2 MPa, in order to force the ionic liquid to flow into the pores of the membrane. After one hour the vessel was opened and the excess ionic liquid remaining on top of the membrane was gently wiped with absorbing tissue. To make sure there was no remaining ionic liquid, the membrane was set upright and left to drip overnight.

### 3.2. Single Gas Permeabilities

**Figure 3:**
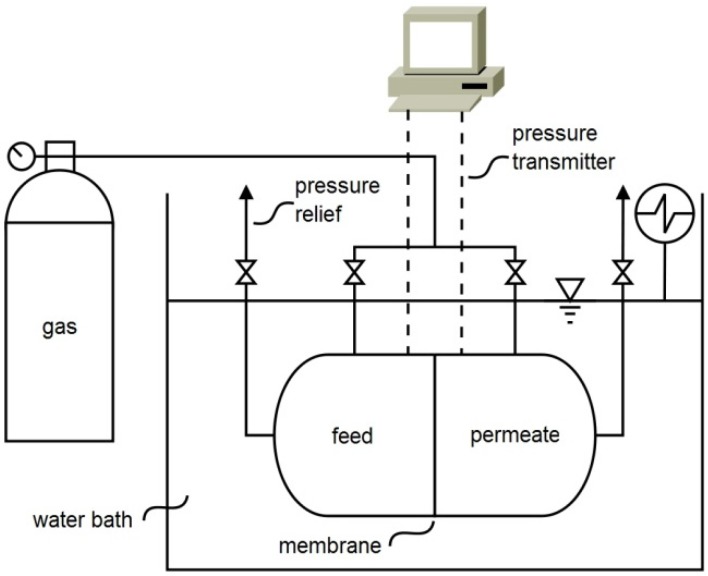
Experimental set-up for measuring the permeability of the membranes for a single gas.

The pure gas permeability of the membrane for N_2_, O_2_ and CO_2_ was determined by using the experimental apparatus shown in Figure 3.

This rig is composed by a stainless steel cell with two identical compartments separated by the membrane. The effective membrane area was 4 cm^2^. Each individual gas permeability was evaluated by pressurizing both compartments (feed and permeate) with the pure gas, and after opening the permeate outlet, establishing a driving force of around 0.07 MPa between the feed and the permeate compartments. The pressure change in both compartments over time was followed using two pressure transducers (Druck, PDCR 910 models 99166 and 991675, Leicester, UK). All measurements were performed at a constant temperature of 303 K by using a thermostatic bath (Julabo, Model EH, Seelbach, Germany). The measurements are reproducible and have an average standard error of 5%.

The permeability of a pure gas through the membrane was calculated from the pressure data measured over time on both compartments (feed and permeate) according to the following equation [33]:

(2) $$\frac{1}{\beta }\text{ln}\;\left(\frac{{\left[p_{\text{feed}}-p_{\text{perm}}\right]}_{0}}{\left[p_{\text{feed}}-p_{\text{perm}}\right]}\right)=\frac{1}{\beta }\ln\;\left(\frac{{\Delta p}_{0}}{\Delta p}\right)=\text{P}\frac{\text{t}}{\text{l}}$$

where p_feed_ and p_perm_ are the pressures in the feed and permeate compartments (Pa), respectively; P is the membrane permeability (m^2^∙s^−1^); t is the time (s); and l is the membrane thickness (m). The indicator 0 refers to the conditions at t = 0. The geometric parameter β (m^−1^) is characteristic of the geometry of the cell and is given by:

(3) $$\beta =A\times (\frac{1}{V_{\text{feed}}}+\frac{1}{V_{\text{perm}}})$$

where A is the membrane area (m^2^) and V_feed_ and V_perm_ are the volumes of the feed and permeate compartments (m^3^), respectively. The β value calculated in this way for the test cell used in this work was 41.97 m^−1^. The data can be plotted as 1/β ln(Δp_0_/Δp) *versus* t/l, and the gas permeability is obtained from the slope of this representation. The ideal selectivity (α_A/B_) is the ratio of the permeabilities of two different pure gases (A and B) through a given membrane.

## 4. Conclusions

A supported ionic liquid membrane was prepared by impregnating [BMIM][DCA] ionic liquid in a PVDF flat sheet membrane, and its permeability to CO_2_, N_2_ and O_2_ was tested and ideal selectivities were calculated. The performance of this membrane was compared to that of Ion-Jelly^®^ membranes and SILMs using PVDF impregnated with other ILs studied in previous works.

The permeability to CO_2_ obtained with this membrane is similar to that obtained with an Ion-Jelly^®^ membrane using the same IL (145 and 120 barrer, respectively) but one order of magnitude lower for N_2_ and O_2_. Consequently, the calculated ideal selectivities are higher for the PVDF membrane impregnated with [BMIM][DCA] studied in this work. The worse performance of the Ion-Jelly^®^ membrane can be a consequence of its higher content in water.

When comparing this membrane with similar membranes using PVDF impregnated with different ILs, it was found that [BMIM][DCA] presents the highest ideal selectivity for the separation of CO_2_ from N_2_ (73), due to a higher diffusion coefficient of CO_2_ in this IL.
